# Association of Higher Advanced Oxidation Protein Products (AOPPs) Levels in Patients with Diabetic and Hypertensive Nephropathy

**DOI:** 10.3390/medicina55100675

**Published:** 2019-10-07

**Authors:** Giovanni Conti, Daniela Caccamo, Rossella Siligato, Guido Gembillo, Ersilia Satta, Dario Pazzano, Nicolina Carucci, Antonio Carella, Giuliana Del Campo, Antonino Salvo, Domenico Santoro

**Affiliations:** 1 Pediatric Nephrology Unit, AOU Policlinic “G Martino”, University of Messina, 98125 Messina, Italy; nicolina88@gmail.com (N.C.); giulianadelcampo@hotmail.it (G.D.C.); 2 Department of Biomedical and Dental Sciences and Morphofunctional Imaging, AOU Policlinic “G Martino”, University of Messina, 98125 Messina, Italy; daniela.caccamo@unime.it (D.C.); antoniocarella9@gmail.com (A.C.); 3 Unit of Nephrology and Dialysis, Department of Clinical and Experimental Medicine, University of Messina, 98125 Messina, Italy; rossellasiligato@gmail.com (R.S.); guidogembillo@live.it (G.G.); ersiliasat@libero.it (E.S.); dario_pazzano@hotmail.it (D.P.); antonino.salvo@polime.it (A.S.); dsantoro@unime.it (D.S.)

**Keywords:** diabetes mellitus, diabetic nephropathy, hypertension, AOPP, albuminuria, CKD, oxidative stress

## Abstract

*Background and Objectives:* Diabetes mellitus (DM) and hypertension (HT) are characterized by cell damage caused by inflammatory and metabolic mechanisms induced by alteration in reduction-oxidative status. Serum advanced oxidation protein products (AOPP) are new markers of protein damage induced by oxidative stress. We evaluated serum levels of AOPP in a cohort of patients with DM and HT, with or without renal complications, compared with a control healthy population. *Materials and Methods:* The study group comprised of 62 patients with type 2 DM and 56 with HT. The 62 patients affected by DM were further distinguished in 24 subjects without renal impairment, 18 with diabetic nephropathy (DN), 20 with chronic kidney disease (CKD) stage 2–3 secondary to DN. The subgroup of 56 patients with primary HT comprised 26 subjects without renal complications and 30 with CKD (stage 2–3) secondary to HT. Thirty healthy controls, matched for age and sex, were recruited among blood donors. *Results:* Increased AOPP levels were found in DM patients compared with healthy subjects, although not significantly. This index was higher and more significant in patients with DN and CKD secondary to DN than in DM patients without nephropathy (*p* < 0.05) or controls (*p* < 0.0001). Patients with HT and with kidney impairment secondary to HT also had significantly higher AOPP serum levels than controls (*p* < 0.01 and *p* < 0.0001, respectively). There were no significant differences in mean AOPP levels among DM and HT patients. *Conclusion:* Our study showed that oxidative stress was higher in diabetic or hypertensive subjects than in healthy controls and, in particular, it appeared to be more severe in patients with renal complications. We suggest that the assessment of AOPP in diabetic and hypertensive patients may be important to predict the onset of renal failure and to open a new perspective on the adoption of antioxidant molecules to prevent CKD in those settings.

## 1. Introduction

End-stage renal disease (ESRD) is a serious health problem in the developed world. Prevalence of ESRD secondary to diabetes mellitus (DM) and hypertension (HT) continues to increase more than by any other cause, as described in various reports in the United States and Europe [1]. Recently, reactive oxygen species (ROS) have been increasingly investigated regarding their role in both pathophysiology and progression of chronic kidney disease (CKD) [2,3]. In general, oxidative stress, including ROS production, can damage cellular macromolecules, leading to DNA and protein alteration, as well as lipid peroxidation. In turn, it causes tissue degeneration, particularly in both micro- and macrovascular systems [4].

Diabetic nephropathy (DN) is one of the major chronic complications of diabetes mellitus and is associated with increased mortality as well as the risk of progression to ESRD, requiring renal replacement therapy [5]. Several studies performed both in vitro and in vivo suggest that oxidative stress is increased in diabetic animal models and patients, and it may contribute to the pathogenesis of diabetic nephropathy [6,7].

Hypertension is another well-known CKD risk factor, even if the mechanisms underlying hypertensive nephropathy are poorly understood. The most accepted hypothesis is mechanical glomerular damage induced by elevated blood pressure, leading to glomerular and arterial sclerosis, infiltration of inflammatory cells, interstitial fibrosis and tubular atrophy, described as “hypertensive nephrosclerosis” [8].

Oxidative stress (OS) is an imbalance between oxidants and antioxidants in favor of antioxidants, leading to a disruption of redox signaling and control and/or molecular damage [9]. OS has been demonstrated to play a major role in different ways, in the global incidence of cardiovascular diseases [10], HT [11], CKD [12], DM [13], dyslipidemia [14], and many other conditions caused by the inflammatory process. Oxidative stress may be a relevant contribution to CKD development, enhancing chronic inflammation and dysregulated apoptotic cell death [15,16], typical of chronic nephropathies, including hypertensive nephrosclerosis [17]. Experimental confirmation of oxidative stress action on blood vessels, and in particular in the kidney, has been shown in many animal models, including spontaneously hypertensive rats [18,19].

Despite advances in our understanding of the pathophysiology underlying OS, there remains a significant need for biomarkers predicting the risk linked to this imbalance of free radical production and antioxidant defenses. Advanced Oxidation Protein Products (AOPP) are regarded as promising markers of oxidant-mediated protein damage. 

AOPPs are dityrosine-containing cross-linked protein products generating albumin and protein oxidation, their production is secondary to OS by the reaction of chlorinated oxidants, with plasma proteins [20,21]. The first time that AOPPs were considered as oxidative stress markers was in 1996 by Witko-Sarsat et al. [22]; on this occasion, AOPPs plasma levels were related to the OS in chronic uremic patients. 

Higher levels of AOPPs have been detected in patients with DM [23,24], cardiovascular diseases [25,26], hypertension [27,28], and atherosclerosis [29].

AOPPs are structurally analogous to advanced glycation end-products (AGEs) and also demonstrated biological activity, as evidenced by their capability to induce pro-inflammatory cytokines and adhesive molecules [30].

Taking these studies into account, our study aimed to evaluate the levels of AOPPs in type 2 DM and HT patients compared with a control healthy population.

## 2. Materials and Methods

Full ethical approval for this study was obtained from the Ethics Committee of the Policlinic G. Martino, University of Messina, Italy (2015-S5536), approved on 15 October 2015.

A total of 118 patients were enrolled in the study compared to a healthy control population.

The first group consisted of 62 patients affected by type 2 DM: 24 of them without diabetic complications, such as nephropathy, neuropathy, hypertension, cardiac ischemic disease, and/or peripheral vasculopathy. Eighteen patients had DN, defined as a persistent albumin excretion rate (200 micrograms/min in sterile urine in at least two of three consecutive assessments), concomitant retinopathy, and no other renal disease or heart failure. Twenty patients were affected by CKD stage 2–3 secondary to DN.

All patients had a diagnosis of type 2 DM, according to the World Health Organization diagnostic criteria. Retinopathy was staged according to Kohner’s classification [31]. All patients were on a low-carbohydrate diet and were treated with insulin. 

The second group consisted of 56 patients with primitive hypertension (HT), none of them was under angiotensin II receptor blockers (ARBs) treatment: 26 HT patients did not have complications, such as nephropathy, neuropathy, cardiac ischemic disease, and peripheral vasculopathy; 30 HT patients had CKD (stage 2–3) secondary to nephroangiosclerosis, without DN.

Estimation of the glomerular filtration rate (eGFR) was computed with the Modification of Diet in Renal Disease (MDRD) formula equation: eGFR (ml/min/1.73 m^2^) = 186.3 × serum creatinine (SCr)^−1.154^ × age^−0.203^ × 0.742 (if female) [32].

We excluded patients affected by acute and chronic infections, fever, malignancy, cirrhosis, and congestive heart failure, as well as acute and chronic nephritis. 

The control group consisted of 30 healthy subjects recruited among blood donors who were referred to our blood transfusion center. None of the control subjects was taking any medication. 

The three main groups of subjects were matched for age and sex. All subjects gave written informed consent to participate in this study in accordance with the Declaration of Helsinki. The characteristics of the study population are shown in Table 1.

### 2.1. Biochemical Assays

After an overnight fast, venous blood samples were collected between 8:00 AM and 9:00 AM through a polyethylene catheter inserted in a forearm vein. All SCr measurements were performed at our chemical clinical laboratory by means of a Colorimetric-kinetic Jaffe reaction with a normal range from 0.5 to 1.4 mg/dL; proteinuria and glucose were measured by colorimetric methods. Insulin was measured by Enzyme ImmunoAssay, with a range of 3–35 µUI/ml. All the components of the kits were stable and stored tightly closed at 2–8 °C, protected from light and contaminations.

#### AOPPs Analysis

The determination of AOPPs plasma levels was carried out by spectrophotometrical measurements using chloramine-T as the standard reference. Briefly, 200 µl of plasma diluted 1:5 in PBS, or chloramine-T standard solutions (0–500 µmol/L), were placed in each well of a 96-well microtiter plate, followed by 20 µl of acetic acid. Ten microliters of 1.16 M potassium iodide (KI) were then added, followed by 20 µl of acetic acid. The absorbance of the reaction mixture was immediately read at 340 nm in a Sunrise^TM^ microplate reader (Tecan Trading AG, Zurich, Switzerland) against a blank containing 200 µl of PBS, 10 µl of KI, and 20 µl of acetic acid. The chloramine-T absorbance at 340 nm was linear within the range of 0–100 µmol/L. AOPPs concentrations were expressed in µmol/liter of chloramine-T equivalents.

### 2.2. Statistical Analysis

Data are presented as mean ± SD. All experimental data in this study were statistically analyzed with SAS 9.13. The statistical significance was evaluated using an unpaired Student’s t-test. Results were considered significant at *p* < 0.05.

## 3. Results

### 3.1. DM Group

The results are described in Table 2. The mean plasma titer of AOPPs was higher in DM patients compared to healthy subjects, but these findings did not reach a statistical significance. We reported, as accessory data, the higher creatinine levels in DM patients, even without any sign of DN, compared to a healthy control population.

Mean plasma levels of AOPPs were significantly higher in DM microalbuminuric patients and those with overt CKD compared to that in DM patients without nephropathy (*p* < 0.05) or in controls (*p* < 0.0001). Duration of DN was longer than that of DM without complications.

Only patients with CKD secondary to DN had significantly higher serum creatinine levels (*p* < 0.05).

### 3.2. HT Group

The results are described in Table 3. The mean plasma levels of AOPPs were significantly higher in HT patients (*p* < 0.01) and in those affected by CKD secondary to HT (*p* < 0.0001) compared to healthy controls. Patients with CKD secondary to HT had significantly longer disease duration and higher serum creatinine levels than HT patients (*p* < 0.05).

No significant differences were observed in mean AOPPs levels among DM and HT patients, with or without complications.

## 4. Discussion

Increased oxidation of proteins, lipids, carbohydrates, and DNA happens when ROS production exceeds local antioxidant capacity. Protein oxidation plays an essential role in the pathogenesis of a large number of degenerative diseases [23].

Several biochemical changes induced by hyperglycemia may, directly and indirectly, influence cellular function, leading to abnormal vascular remodeling and contributing to the development of other diabetic complications as DN [33,34,35]. High glucose concentrations are associated with ROS formation and oxidative stress by different molecular mechanisms, such as an increased flux through the polyol and glucosamine pathways, activation of protein kinase C and NADPH oxidase, as well as the production of AGEs [3,36,37,38].

Release of AGEs, production of reactive oxygen species (ROS), glycosylation of glomerular and tubular proteins with activation of Janus kinase/signal transducers/activators of transcription (JAK-STAT), nuclear factor-kB (NFkB), and p38 mitogen-activated protein kinase pathways are some of the hypothesized mechanism leading to DN and CKD secondary to DN. In addition, an increase in the concentration of cytokines and pro-fibrotic proteins could contribute to these conditions [39,40,41].

Amore et al. [42] showed in vivo modulation of NFkB activation by N-acetyl-cysteine in DM patients on hemodialysis, suggesting a potential clinical benefit for the inhibition of oxidative stress pathways.

As well as it knows, hypertension maintains oxidative stress in large blood vessels and highly vascularized organs, especially the kidney [43]. Oxidative stress has many possible consequences: it stimulates growth-signaling pathways, induces expression of pro-inflammatory genes, and has a role in impairing endothelial function [44,45]. Among possible mediators of the oxidative stress, angiotensin II has been singled out, both in the kidney and other organs in vivo [46,47,48]. Angiotensin II could also contribute to apoptosis by oxidative stress mechanisms in cultured mesangial cells [48].

Data suggest that chronic accumulation of AOPPs in plasma may promote renal inflammation in DM patients probably through activation of renal NADPH oxidase and is associated with increased susceptibility to develop diabetic retinopathy [49]. The concentration of AOPPs reflects plasma proteins’ oxidation, especially albumin [41].

Li et al. [50] enlightened the link between AOPPs and endothelial-to-mesenchymal transition on human renal glomerular endothelial cells, leading to the worsening of DN due to the endoplasmic reticulum stress. Also, Rong et al. [51] demonstrated endoplasmic reticulum stress and podocyte apoptosis secondary to the accumulation of AOPPs.

In a study on DN patients and diabetic mice in vivo, Li et al. [50] demonstrated the role of AOPPs and their receptor CD 36 in lipotoxicity, lipid accumulation, and tubulointerstitial fibrosis, all leading to worst outcomes of the comorbidities of these subjects. 

More studies on rodents demonstrated experimentally the role of AOPPs in promoting redox-sensitive inflammatory pathways, which contribute to the pathogenesis of glomerulosclerosis. Intravenous administration of AOPPs in diabetic rats demonstrated their higher concentration in kidney promoted inflammation, glomerular hypertrophy, and overexpression of fibronectin, leading to albuminuria [52].

In our study, we investigated oxidative stress status in type 2 DM and hypertensive patients, whether any difference exists between DM and HT with and without renal complications. 

For this purpose, we analyzed the plasma levels of AOPPs in patients at different stages of DM (without complications, with microalbuminuric DN or with overt CKD) and in a group of patients affected by several degrees of hypertension (without complications or affected by CKD). 

Our data confirmed those of Pan et al. [52] who demonstrated greater oxidative stress in DN patients compared to those with DM without complications. Liang et al. [53] showed that increased plasma AOPPs concentrations were an independent risk factor for endothelial dysfunction in DM patients without albuminuria. The evidence that oxidative stress is involved in the pathogenesis of DN promotes the hypothesis on the use of new drugs in prevention and treatment, such as pyridoxine, bardoxolone, GLY-230, and others [40]. 

The present trial indicates that a close linkage may exist between higher AOPPs levels and both DN and hypertensive patients with CKD, compared to the healthy control group.

## 5. Conclusions

Notwithstanding higher AOPPs levels detected in patients with DM and HT than in the control group, statistical significance was reached comparing patients affected by CKD secondary to either DM and HT and healthy subjects. Until now, the measurement of urinary albumin excretion rate and plasma creatinine concentration are the most reliable indicators of glomerular injury. Besides these biomarkers, the measurement of oxidative stress levels with AOPPs seems to be a promising new indicator of renal impairment, especially in DN and HT. According to our single-center experience, we suggest that the assessment of oxidative stress in diabetic and hypertensive patients may be a predictive factor to assess the progression of kidney damage, but further studies with larger cohorts are needed to encourage a potential clinical use of AOPPs and to investigate their pathogenic role.

## Figures and Tables

**Table 1:** Clinical characteristics of diabetes mellitus and hypertension patients and a healthy control group.

Clinical Characteristics	Healthy Control Group (HCG)	Diabetes Mellitus (DM)	Diabetic Nephropathy (DN)	Chronic Renal Failure for DN	Hypertension (HT)	Chronic Renal Failure for HT
*n*	30	24	18	20	26	30
Age (years)	53.83 ± 12.65	51.85 ± 13.46	52.65 ± 12.28	54.12 ± 11.75	53.28 ± 13.72	52.91 ± 11.45
Sex (M/F)	13/17	10/14	8/10	9/11	11/15	14/16
Serum Glucose (mmol/l)	4.76 ± 0.39	10.38 ± 2.41 ^a^	11.55 ± 2.85 ^a^	11.03 ± 3.08 ^a^	4.58 ± 0.28	4.97 ± 0.45
Insulin (mmol/l)	-	11.39 ± 8.75	10.66 ± 6.09	11.69 ± 7.61	-	-
Systolic Blood Pressure (mmHg)	130.2 ± 6.58	132.45 ± 8.45	133.48 ± 9.5	137.5 ± 10.6 ^a^	148.85 ± 8.3 ^a^	149.5 ± 7.6 ^a^
Diastolic Blood Pressure (mmHg)	75.68 ± 8.8	77.42 ± 6.8	78.21 ± 7.25	84.3 ± 5.65 ^a^	80.85 ± 7.55	85.451 ± 8.05

Data are mean values ± SD; ^a^ *p* < 0.01 vs. HCG.

**Table 2:** Plasma levels of the advanced oxidation protein product (AOPP) in diabetes mellitus patients without or with renal complications compared with a healthy control group.

Variables Considered in HCG and Diabetic Patients	Healthy Control Group (HCG)	Diabetes Mellitus (DM)	Diabetic Nephropathy (DN)	Chronic Renal Failure for DN
*n*	30	24	18	20
Plasma levels of AOPP (micromole/l)	124 ± 47	155 ± 110	252 ± 163 ^a^	225 ± 95 ^a^
Duration of the disease (months)	-	31.05 ± 32.15	69.83 ± 41.18 ^b^	75.43 ± 52.85 ^b^
Serum creatinine (micromole/l)	58.85 ± 12.82	63.73 ± 11.06	65.11 ± 12.23	77.35 ± 21.08 ^c^
Urinary albumin excretion rate (microgram/min)	<20	<20	>200	>200

Data are mean values ± SD; ^a^ *p* < 0.05 vs. DM and *p* < 0.0001 vs. HCG; ^b^ *p* < 0.01 vs. DM; ^c^ *p* < 0.05 vs. HCG, DM, and DN.

**Table 3:** Plasma levels of AOPP in hypertension patients without or with renal complications compared with a healthy control group.

Variables Considered in HCG and Hypertension Patients	Healthy Group Control (HCG)	HT	Chronic Renal Failure for HT
*n*	30	26	30
Plasma levels of AOPP (micromole/l)	124 ± 47	175 ± 97 ^a^	217 ± 110 ^b^
Duration of the disease (months)	-	33.89 ± 31.75	70.03 ± 48.58 ^c^
Serum creatinine (micromole/l)	58.85 ± 12.82	64.22 ± 14.53	74.7 ± 18.81 ^d^

Data are mean values ± SD; *n* = numbers, ^a^ *p* < 0.01 vs. HCG; ^b^ *p* < 0.0001 vs. HCG ^c^ *p* < 0.05 vs. HT; ^d^ *p* < 0.05 vs. HCG and HT.
